# The Inhibition of Cathepsin G on Endometrial Explants With Endometrosis in the Mare

**DOI:** 10.3389/fvets.2020.582211

**Published:** 2020-10-30

**Authors:** Ana Amaral, Carina Fernandes, Sofia Morazzo, Maria Rosa Rebordão, Anna Szóstek-Mioduchowska, Karolina Lukasik, Barbara Gawronska-Kozak, Luís Telo da Gama, Dariusz Jan Skarzynski, Graça Ferreira-Dias

**Affiliations:** ^1^Department Morfologia e Função, Faculdade de Medicina Veterinária, CIISA—Centro de Investigação Interdisciplinar em Sanidade Animal, Universidade de Lisboa, Lisboa, Portugal; ^2^Polytechnic of Coimbra, Coimbra Agriculture School, Coimbra, Portugal; ^3^Institute of Animal Reproduction and Food Research, Polish Academy of Science, Olsztyn, Poland

**Keywords:** endometrosis, cathepsin G, cathepsin G inhibitor, fibrosis, metallopeptidases

## Abstract

Although proteases found in neutrophil extracellular traps (NETs) have antimicrobial properties, they also stimulate collagen type 1 (COL1) production by the mare endometrium, contributing for the development of endometrosis. Cathepsin G (CAT), a protease present in NETs, is inhibited by specific inhibitors, such as cathepsin G inhibitor I (INH; β-keto-phosphonic acid). Matrix metallopeptidases (MMPs) are proteases involved in the equilibrium of the extracellular matrix. The objective of this study was to investigate the effect of CAT and INH (a selective CAT inhibitor) on the expression of MMP-2 and MMP-9 and on gelatinolytic activity. In addition, the putative inhibitory effect of INH on CAT-induced COL1 production in mare endometrium was assessed. Endometrial explants retrieved from mares in follicular phase or midluteal phase were treated for 24 or 48 h with CAT, inhibitor alone, or both treatments. In explants, transcripts (quantitative polymerase chain reaction) of *COL1A2, MMP2*, and *MMP9*, as well as the relative abundance of COL1 protein (Western blot), and activity of MMP-2 and MMP-9 (zymography) were evaluated. The protease CAT induced COL1 expression in explants, at both estrous cycle phases and treatment times. The inhibitory effect of INH was observed on *COL1A2* transcripts in follicular phase at 24-h treatment, and in midluteal phase at 48 h (*P* < 0.05), and on the relative abundance of COL protein in follicular phase and midluteal phase explants, at 48 h (*P* < 0.001). Our study suggests that MMP-2 might also be involved in an earlier response to CAT, and MMP-9 in a later response, mainly in the follicular phase. While the use of INH reduced CAT-induced COL1 endometrial expression, MMPs might be involved in the fibrogenic response to CAT. Therefore, in mare endometrium, the use of INH may be a future potential therapeutic means to reduce CAT-induced COL1 formation and to hamper endometrosis establishment.

## Introduction

In the endometrium, the innate and adaptive immune mechanisms, which rely on a complex network of key components (mainly growth factors/cytokines, immune cells, and epithelial and stromal cells), modulate integrated interactions between the endocrine system and the immune system. As such, they regulate uterine physiological function and provide protection against pathogens (1–3). Disruption of those immune-endocrine mediated mechanisms may lead to endometrial dysfunction and ultimately to fibrogenesis and infertility (2, 3).

A transient breeding-induced endometritis is a normal process to remove bacteria and the excess of spermatozoa from the uterus, causing an increase of neutrophils influx to the uterine lumen, which in turn increases the uterine inflammatory reaction (4–6). If the inflammation becomes chronic, the persistent influx of neutrophils toward the endometrium prompts to chronic degenerative alterations, ending in endometrosis (endometrial fibrosis) (7). However, impaired uterine clearance (6), repeated endometritis (8), and aging and multiple pregnancies (9) have been described as triggering factors of equine endometrosis. Equine endometrial fibrosis is a progressive and irreversible severe fibrotic disorder in the endometrium (7, 10, 11), causing subfertility/infertility. At the initial stage of endometrosis, fibroblasts differentiate into myofibroblasts responsible for the synthesis of collagen fibers and extracellular matrix deposition, ultimately leading to endometrial periglandular fibrosis (7, 12). Thus, these histological changes are the culprit of a decrease in pregnancy rates in the mare (10, 13).

The presence of bacteria or semen in the equine endometrium (14–16) induces neutrophil migration from blood to the uterus to fight the infection. These neutrophils release proteins and components from the nucleus that form “neutrophil extracellular traps” (NETs) extracellularly (14, 16, 17). Proteases present in NETs, namely, cathepsin G (CAT), elastase (ELA), or myeloperoxidase (MPO), possess strong antimicrobial properties, aiding on killing bacteria in the extracellular environment. However, their persistence may lead to chronic inflammation and degenerative changes in equine endometrium (18). Increased collagen type 1 (COL1) in mare endometrial explants challenged with NET components has been described previously (18–20).

Cathepsin G participates to a greater extent to inflammation and fibrosis establishment in chronic obstructive pulmonary disease (COPD) in humans (21). Also, CAT action was associated with aortic stenosis remodeling and fibrosis (22), renal fibrosis after ischemia (23) glomerulonephritis and renal failure (24), lung cystic fibrosis [(25), reviewed by (26), (27)], and fibrotic Dupuytren disease in humans (28). Cathepsin G inhibitor I (β-keto-phosphonic acid; INH) is a small non-peptide molecule that in a selective, potent, and reversible manner inhibits CAT. This inhibitor could be used for the treatment of COPD and asthma in humans (21, 29, 30). Additionally, INH exhibits an anti-inflammatory action in rats with glycogen-induced peritonitis and lipopolysaccharide-induced inflammation of the airways (29) and in airway inflammatory diseases dependent on CAT in animal models (30).

Matrix metallopeptidases (MMPs) are involved in extracellular matrix balance and in endometrial tissue remodeling (31). These enzymes have the capability to degrade extracellular matrix structural components, such as collagen (32). In the equine endometrium, during bacterial and breeding-induced acute endometritis, MMP-2 and MMP-9 are engaged in the inflammatory reaction and COL modification (33). But, if an alteration in the regulation of these MMPs or a prolonged exposure to inflammation occurs, it leads to deposition of COL and subsequent establishment of endometrial fibrosis (33). In our recent *in vitro* studies on equine endometrium, MMP expression was affected by mediators of inflammation, such as interleukins, transforming growth factor β1 (TGFβ1), and prostaglandins (PGs) (12, 34, 35); differs among stages of endometrosis (35); and might be implicated in fibrotic response to ELA (20).

It has been known that ELA and CAT proteases released by neutrophils are capable of destroying the extracellular matrix, stimulating leukocyte migration, and inducing tissue remodeling (29, 36). Our previous *in vitro* studies reported them as being also associated with endometrial fibrosis establishment (18–20). In fact, ELA, CAT, and MPO appear to act as profibrotic factors in mare endometrosis (18). The inhibition of ELA using sivelestat sodium salt, a specific ELA inhibitor, provoked a downregulation of *COL1A2* mRNA transcription (18, 20). Among proteases present in NETs, the one that shows the predominant proteolytic activity is ELA. Nevertheless, when ELA was immune depleted from NETs derived from healthy human neutrophils, the remaining activity was attributed to CAT (37). Moreover, in the pathophysiology of COPD in humans, CAT seems to play a particularly important role (29). These findings justify the recent development of diagnostic tests that use CAT as a COPD marker (38). Thus, the importance of studying inhibitors of proteases present in NETs, such as CAT, is imperative for the development of putative therapeutic measures for the control of fibrosis.

Because CAT (present in NETs) and MMPs appear to be involved in the development of equine endometrosis (18, 20), we have decided to investigate potential putative ways of fighting this condition by impairing fibrosis formation. We hypothesized that by inhibiting CAT using a specific inhibitor (β-keto-phosphonic acid), it would be possible to reduce the COL1 output and thus hinder the profibrotic response to CAT in equine endometrial explants. Therefore, the objective of this *in vitro* study was to investigate the INH inhibitory action on the relative abundance of CAT-induced COL1 protein in explants of mare endometrium. In addition, the influence of CAT and INH on MMP-2 and MMP-9 expression and gelatinolytic activity was assessed.

## Materials and Methods

### Animals

From April to September, at an abattoir in Poland (Rawicz), uteri and jugular venous blood were randomly retrieved postmortem from cyclic mares destined for meat production, according to the European (European Food Safety Authority, AHAW/04-027) legislation. Mares' average age was 12 years. The official veterinary inspection certified that those mares were healthy, and their meat was safe for human consumption. Estrous cycle phase of each mare was determined based on ovarian and uterine features and on progesterone plasma concentration, as previously described (18, 39). Thus, mares, which presented a follicle >35-mm diameter, absence of an active corpus luteum, and plasma progesterone concentration <1 ng/mL, were classified as being in the follicular phase. In contrast, the existence of a well-developed corpus luteum associated with the presence of follicles with a diameter between 15 and 20 mm and plasma progesterone concentration >6 ng/mL were the grounds for considering those mares in the midluteal phase. For the present study, follicular phase (*n* = 8) and midluteal phase (*n* = 7) endometria were used. After collection, jugular venous blood in ethylenediaminetetraacetic acid tubes and uteri were transported on ice to the laboratory. The uteri were placed in ice-cold Dulbecco modified Eagle medium (DMEM) F-12 Ham medium (D/F medium; 1:1 (vol/vol); D-2960; Sigma–Aldrich, St. Louis, MO, USA), supplemented with antibiotics, such as penicillin (100 IU/mL; P3032; Sigma–Aldrich) and streptomycin (100 μg/mL; S9137; Sigma–Aldrich), and an antimycotic drug, amphotericin (2 μg /mL; A2942; Sigma–Aldrich). All the mares' uteri used were examined for the absence of endometritis, both macroscopically and microscopically. The macroscopic examination enabled the visualization of increased mucus production or altered endometrial surface color in the presence of endometritis. The microscopic evaluation of the eventual presence of bacteria and/or neutrophils in the endometrium was accomplished by collecting the cells with a sterile swab, rolled on a glass slide, and colored with Diff-Quick stain (18, 40). Endometritis was the grounds for discarding the uteri. To perform the histological and endometrial classification (41), two fresh endometrial samples (around 0.5**-**cm width by 2**-**cm length), immediately after collection, from each uterus were immersed in 4% buffered paraformaldehyde. Changes in mare endometrium were assessed as described by Kenney and Doig (41). Regarding the amount of endometrial inflammation and/or fibrosis, endometria were classified as I, IIA, IIB, or III categories, according to Kenney and Doig (41). Slight to scattered inflammation (endometritis) or mild fibrosis (endometrosis) or mild lymphatic lacunae can be found in Kenney and Doig's category IIA. In category IIB, there might be moderate inflammation, but mostly moderate fibrosis that can be multifocal or diffuse, or moderate lymphatic lacunae (41, 42), although, in this study, only endometria with mild to moderate fibrotic lesions (IIA or IIB category) were used, avoiding endometria with inflammation (endometritis). Besides, no category III endometria were used to exclude possible variations due to increased endometrial fibrotic lesions.

### *In vitro* Endometrial Explant Culture

Strips (around 0.5-cm width by 2- to 3-cm length) of endometrium from the ipsilateral horn to the active ovary were detached from the myometrium after the uterus was washed in phosphate-buffered saline (PBS) with streptomycin (100 μg /mL; S9137; Sigma–Aldrich) and penicillin (100 IU/mL; P3032; Sigma–Aldrich) added.

For explant culture experiments, strips of endometrium were put in ice-cold PBS supplemented with antibiotics (as above) in a Petri dish. Then, the endometrium strips were washed with PBS supplemented with antibiotics and endometrial explants, cut, and blotted with filter paper. The explants, weighing from 20 to 30 mg each, were placed in a single well of a sterile 24-well cell culture plate (Eppendorf, #0030 722.116) with 1 mL of DMEM culture medium with bovine serum albumin (0.1% wt/vol; 735078; Roche Diagnostics, Mannheim, Germany), streptomycin (100 μg/mL; S9137; Sigma–Aldrich), penicillin (100 IU/mL; P3032; Sigma–Aldrich), and amphotericin (2 μg/mL; A2942; Sigma–Aldrich). The endometrial explants were preincubated at 38°C, in a 5% CO_2_ humidified atmosphere (Biosafe Eco-Integra Biosciences, Chur, Switzerland), for 1 h, and submitted to 150 rpm gentle shaking, as described previously (18). Afterward, culture medium was replaced, and equine endometrial explants were treated for 24 or 48 h, as follows: (i) vehicle (negative control)—culture medium alone; (ii) CAT (1 μg/mL; A6942, Applichem GmbH, Germany); (iii) cathepsin G inhibitor I (INH; 1 μg/mL; β-keto-phosphonic acid; C_36_H_33_N_2_O_6_P, sc-221399; Santa Cruz Biotechnology, USA); and (iv) CAT (1 μg/mL) + INH (1 μg/mL). Each treatment was performed in quadruplicate. The INH was added after 1 h of preincubation, at the time of culture medium replacement, to allow time for the inhibitor to bind. Protease CAT was added 1 h later. In studies undergoing a total of 48 h, after 24-h treatment, 1 μg/mL of IHN was added once again to the culture medium, because in the pretrial its inhibitory effect remained for only 24 h and waned at 48-h treatment. At the end of each treatment time, explants were collected and placed in RNAlater (R901, Sigma–Aldrich), while conditioned media were collected and stored at −80°C. In a previous study, as a positive control for COL expression, endometrial tissue response to a fibrotic stimulus was assessed by adding TGFβ1 (a profibrotic cytokine) to tissue culture medium (20). To assess viability, the explants were also incubated with oxytocin (OXT), as described before (20).

As shown by our previous work, when dose assessment was determined (18), the use of 1 μg/mL of CAT proved to induce the expression of fibrotic marker, TGFβ1. A dose–response pilot experiment was performed to assess the most suitable concentration of INH, based in other previous *in vitro* studies (43). The INH was tested using 0.01, 0.1, 1, 10, and 100 μg/mL, and the optimal concentration that inhibited *COL1A2* transcription was 1 μg/mL (data not shown).

### Assessment of Endometrial Explants Viability

The assessment of endometrial explant viability was based on lactate dehydrogenase (LDH) activity as described before (20) and on OXT-induced PGF_2α_ secretion in conditioned culture medium. The PGF_2α_ secretion was determined by using an enzyme immunoassay kit (ADI-901-069, Enzo), according to the manufacturer's instructions.

### Quantitative Real-Time Polymerase Chain Reaction

Total RNA from equine endometrial treated explants was extracted using TRI Reagent® (T9424; Sigma–Aldrich), as indicated by the manufacturer. After, RNA quantification and quality evaluation were performed, as described previously (20). Specific primers for the reference gene ribosomal protein L32 (*RPL32*) and for *COL1A2, MMP2*, and *MMP9* are presented in Table 1. The reference gene *RPL32* was the most stable internal control, already determined in a previous study (20, 44). All the reactions for target and reference genes were performed in duplicate, on a 96 well plate (4306737; Applied Biosystems) and run in a StepOnePlus™ Real-Time PCR System (Applied Biosystems, Warrington, UK). To confirm specificity, the polymerase chain reaction (PCR) products were run on a 2.5% agarose gel, and relative mRNA data were quantified using the quantitative PCR miner algorithm. Briefly, the average of the cyclic threshold (Cq) and the primer efficiency level (E) for each sample were related using the following equation: [1/(1þE)Cq]. Afterward, the expression levels of the target genes were normalized against the reference gene (45).

**Table 1:** Primers used in quantitative PCR.

**Gene (accession number)**	**Sequence 5^**′**^-3^**′**^**	**Amplicon**
*COL1A2* (XM_001492939.3)	Forward: CAAGGGCATTAGGGGACACA	196
	Reverse: ACCCACACTTCCATCGCTTC	
*MMP2* (XM_001493281.2)	Forward: TCCCACTTTGATGACGACGA	115
	Reverse: TTGCCGTTGAAGAGGAAAGG	
*MMP9* (NM_001111302.1)	Forward: GCGGTAAGGTGCTGCTGTTC	177
	Reverse: GAAGCGGTCCTGGGAGAAGT	
*RPL32* (XM_001492042.6)	Forward: AGCCATCTACTCGGCGTCA	144
	Reverse: GTCAATGCCTCTGGGTTTCC	

*COL1A2, collagen type 1 α2; MMP2, matrix metallopeptidase 2; MMP9, matrix metallopeptidase 9; RPL32, ribosomal protein L32*.

### Western Blot Analysis

Protein relative abundance of COL1 was determined by Western blot technique using a non-staining total protein loading control as previously described (20, 46). The membranes were incubated overnight, at 4°C with the primary antibody against COL1 (1:1,000 diluted; 20121; Novotec, Lyon, France), as previously defined (18). The secondary antibody used was horseradish peroxidase–conjugated anti-rabbit (1:20,000; P0448; DakoCytomation, Carpinteria, CA, USA) incubated at room temperature for 1.5 h. Visualization of the relative abundance of COL1 protein was accomplished by luminol enhanced chemiluminescence (Super Signal West Pico, 34077; Thermo Scientific, Waltham, MA, USA). For band normalization in each membrane, and to allow band comparison between membranes, a standard sample of a blend of endometrial explants (30 μg) was loaded in a single lane, in all gels. Image acquisition and band normalization were performed, as described (20, 47).

### Zymography

The activity of MMP-2 and MMP-9 on gelatin gel was assessed by zymography, through a non-staining total protein loading control, as previously described (20, 48, 49). Gels and samples of culture medium supernatant were handled, as referred by Amaral et al. (20). In all gels, molecular weight determination was made using recombinant human MMP-2 protein, CF (902-MP-010; R&D Systems, Minneapolis, USA) and recombinant human MMP-9 Western Blot Standard Protein (WBC018; R&D Systems). In order to normalize and compare gels, a standard sample (40 μg) of mixed culture medium was also loaded. The images detection and MMPs gelatinolytic activity were determined as already reported (20). Briefly, using Image Lab 6.0 (Bio-Rad) software, the lanes were detected in a non-staining total protein gel image, and the bands corresponding to MMP-2 and MMP-9 activity were detected on the Coomassie staining image. The normalization factor and volume of target protein were calculated by the software, and then the values were adjusted for variation in the protein load.

### Statistical Analysis

**Figure 1:**
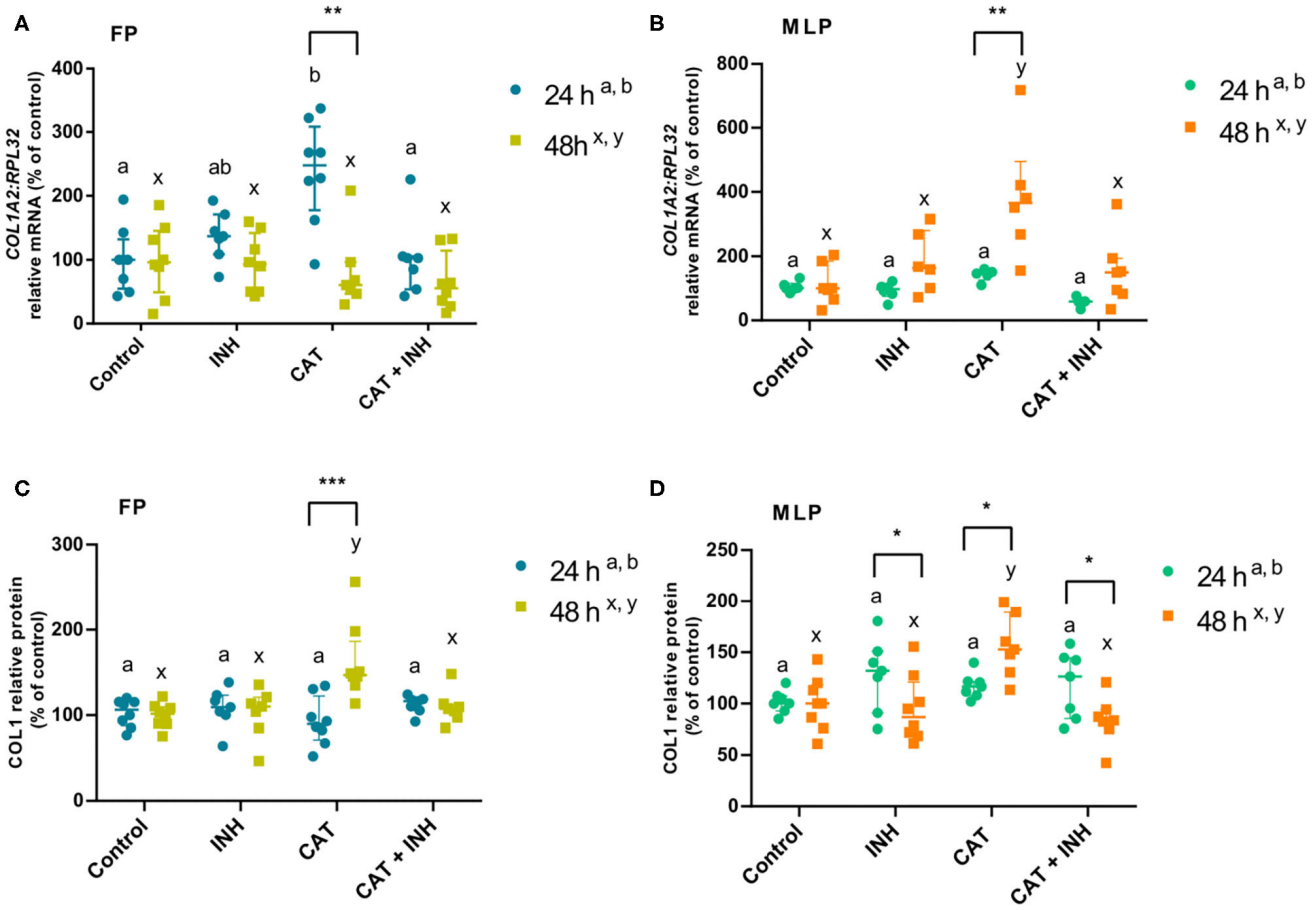
Relative collagen type 1 (*COL1A2*) mRNA transcription **(A,B)** and protein (COL1) relative abundance **(C,D)** in follicular phase (FP) and midluteal phase (MLP) mare endometrial explants treated for 24 or 48 h with culture medium alone (control), cathepsin G inhibitor I (INH: 1 μg/mL), cathepsin G (CAT: 1 μg/mL), or CAT (1 μg/mL) + INH (1 μg/mL). Data are shown as median with interquartile range. Results were considered significant at *P* < 0.05. Different superscript letters indicate significant differences between treatments within each treatment time (a,b: 24 h; x,y: 48 h). Asterisks indicate statistical differences between times of treatment for the same treatment (*P < 0.05, **P < 0.01, ***P < 0.001).

**Figure 2:**
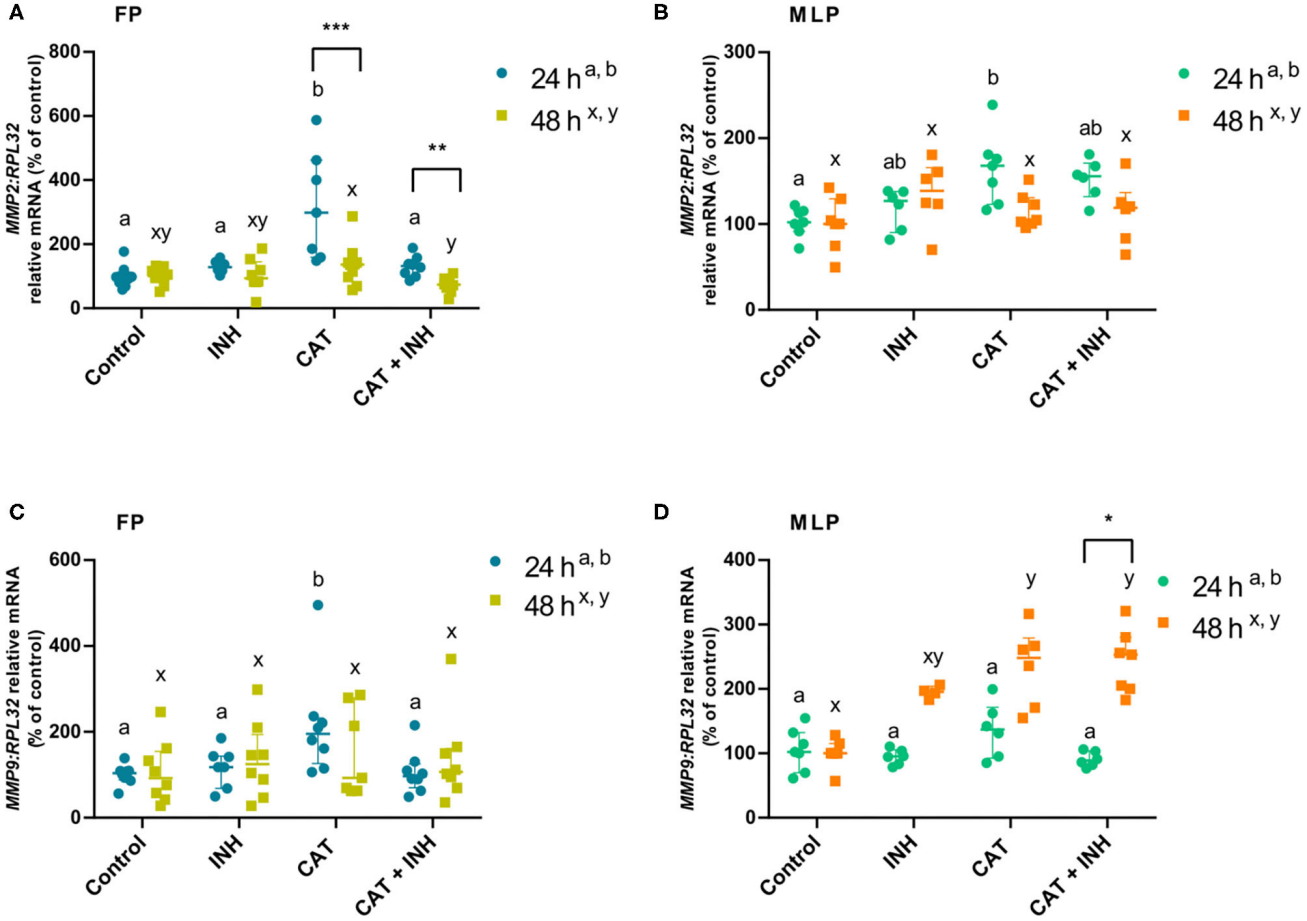
Relative mRNA transcription of *MMP2* **(A,B)** and *MMP9* **(C,D)** in follicular phase (FP) and midluteal phase (MLP) mare endometrial explants treated for 24 or 48 h with culture medium alone (control), cathepsin G inhibitor I (INH: 1 μg/mL), cathepsin G (CAT: 1 μg/mL), or CAT (1 μg/mL) + INH (1 μg/mL). Data are shown as median with interquartile range. Results were considered significant at *P* < 0.05. Different superscript letters indicate significant differences between treatments within each treatment time (a,b: 24 h; x,y: 48 h). Asterisks indicate statistical differences between times of treatment for the same treatment (*P < 0.05, **P < 0.01, ***P < 0.001).

**Figure 3:**
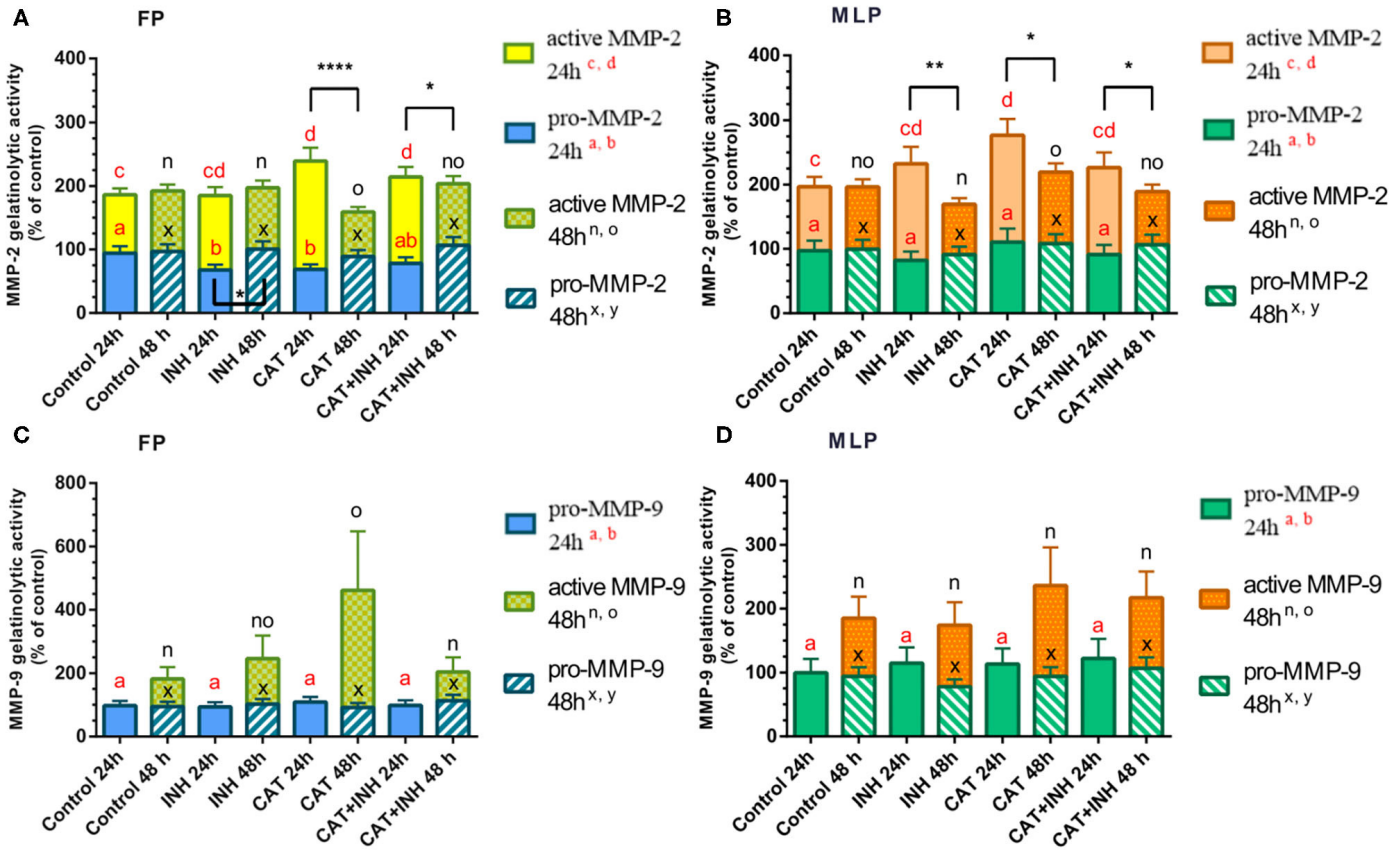
Relative gelatinolytic activities of MMP-2 **(A,B)** and MMP-9 **(C,D)** in follicular phase (FP) and midluteal phase (MLP) mare endometrial explants treated for 24 or 48 h with culture medium alone (control), cathepsin G inhibitor I (INH: 1 μg/mL), cathepsin G (CAT: 1 μg/mL), or CAT (1 μg/mL) + INH (1 μg/mL). All values are expressed as percentage of change from control (non-treated tissues). Bars represent least square means ± SEM, and results were considered significant at *P* < 0.05. Different superscript letters indicate significant differences between treatments within each treatment time. Asterisks indicate statistical differences between different treatment times for the same treatment and for the same form of MMP (*P < 0.05, **P < 0.01, ****P < 0.0001).

The variables assessed in this study were *COL1A2, MMP2*, and *MMP9* transcription; COL1 protein relative abundance; and gelatinolytic activity of both proactive and active forms of MMP-2 and MMP-9. The Kolmogorov–Smirnov test in Proc Univariate function of SAS v. 9.4 (SAS Institute Inc.) and visual examination were used to check data normality. The square root and logarithmic transformations were achieved because some of the variables did not show a normal distribution, and the best transformation method was chosen. At first, the response variables were analyzed by PROC GLM of SAS, as a function of the different treatments: combination of the use of CAT, use of INH, estrous cycle phase, and incubation time, in a total of 16 treatment combinations. Using the PDIFF option of PROC GLM, the least square means of the treatments combinations were compared, and the results were considered significant as *P* < 0.05. For data plotting, the means were back transformed to the original scale. Afterward, the two-, three-, and four-way interactions of the treatment combinations were also performed. In Figures 1, 2, the results of relative abundance of COL1 protein and *COL1A2, MMP2*, and *MMP9* transcripts are depicted as median with interquartile range. In Figure 3, gelatinolytic activity data for MMP-2 and MMP-9 are shown as least square means ± SEM. The graphs presented were built using GraphPad PRISM.

## Results

### Long-Term Viability of Explants From Equine Endometrium

As shown before by Amaral et al. (20), *COL1A2* transcription and protein relative abundance of COL1 were upregulated in response to TGFβ1 treatment. About viability data, no difference was found in LDH activity between 1- and 24-h treatment times, but a slight decrease was shown at 48 h, regardless of estrous cycle phase. Besides, mare endometrial tissues treated with OXT augmented PGF_2α_ secretion at both estrous cycle phases and treatment times (Supplementary Table 1).

### The Effect of INH on CAT-Induced COL1

Supplementary Table 2 lists the interactions among treatments, time of treatment, and estrous cycle phase. The differences between estrous cycle phases (follicular phase vs. midluteal phase) within each treatment and treatment times are presented in Supplementary Table 3.

The treatment with CAT elevated *COL1A2* transcripts in follicular phase endometrial explants at 24 h (*P* < 0.01; Figure 1A) and in midluteal phase tissue at 48 h (*P* < 0.0001; Figure 1B) relative to the respective control group. Nevertheless, the combination of CAT and INH downregulated *COL1A2* transcripts compared to the corresponding CAT-treated groups (follicular phase 24 h: *P* < 0.01; midluteal phase 48 h: *P* < 0.001; Figures 1A,B). In midluteal phase, at 48 h, the transcription also increased in CAT-treated explants regarding INH-treated group (*P* < 0.001; Figure 1B).

In CAT-treated tissues, COL1 protein relative abundance increased in the longest period of treatment both in follicular phase (*P* < 0.01; Figure 1C) and midluteal phase explants (*P* < 0.001; Figure 1D, Supplementary Figure 1) relative to the control group. The association of CAT and INH reduced protein relative abundance after 48-h treatment both in follicular phase (*P* < 0.01; Figure 1C) and midluteal phase explants (*P* < 0.001; Figure 1D, Supplementary Figure 1) compared to the respective CAT-treated groups. Explants treated with CAT also elevated COL protein relative abundance at 48 h, both in follicular phase (*P* < 0.01; Figure 1C), and in midluteal phase endometria (*P* < 0.001; Figure 1D, Supplementary Figure 1), when compared to the respective INH-treated group.

At 24 h, in follicular phase, in the CAT-treated group, *COL1A2* mRNA transcription was higher when compared to 48 h (Figure 1A), although the protein relative abundance was higher at 48 h (Figure 1C). But, in midluteal phase tissues, CAT treatment upregulated *COL1A2* transcripts (Figure 1B) and COL1 protein relative abundance (Figure 1D) at 48 h when compared to 24 h. Also, in midluteal phase at 48 h, the COL1 protein relative abundance was reduced in INH-treated and CAT+INH–treated groups when compared to 24-h treatment (Figure 1D, Supplementary Figure 1).

### Evaluation of CAT and INH Effect on MMP Expression

The *MMP2* transcript levels increased in CAT-treated explants in follicular phase at 24 h compared to its respective control group (*P* < 0.001) and INH group (*P* < 0.05; Figure 2A). But, when those explants were submitted to the combination of CAT and INH, there was a reduction in *MMP2* mRNA, comparing to the respective CAT-treated tissues (*P* < 0.01; Figure 2A). In the same estrous cycle phase, but after 48-h treatment, CAT+INH treatment reduced *MMP2* transcripts in relation to the CAT-treated group (*P* < 0.01; Figure 2A), which was not increased when compared to control. In midluteal phase explants, at 24 h, CAT treatment augmented *MMP2* mRNA when related to the respective control (*P* < 0.05; Figure 2B).

In the follicular phase, at 24 h, the CAT treatment was able to increase *MMP9* mRNA levels in endometrial explants with respect to the respective control group (*P* < 0.01; Figure 2C) and INH-treated group (*P* < 0.05; Figure 2C). However, the CAT+INH–treated explants reduced *MMP9* transcripts compared to the CAT-treated group (*P* < 0.05; Figure 2C). At 48 h, midluteal phase endometrium treated with CAT upregulated *MMP9* transcription (*P* < 0.05), which further increased with CAT+INH treatment (*P* < 0.01; Figure 2D) compared to the non-treated group.

In follicular phase, the treatments of CAT and CAT+INH increased *MMP2* transcripts at 24 h in comparison to 48 h (Figure 2A). In contrast, in midluteal phase endometrium, in explants treated for 48 h, the combination of CAT and INH augmented *MMP9* transcripts with respect to 24-h treatment (*P* < 0.05; Figure 2D).

The analysis of the proform of MMP-2 gelatinolytic activity has shown that INH-treated and CAT-treated groups decreased its activity in follicular phase at 24 h (*P* < 0.05; Figure 3A, Supplementary Figure 1). Nevertheless, in follicular phase endometrial explants treated for 24 h with CAT and combination of CAT+INH, the gelatinolytic activity of MMP-2 active form was upregulated with respect to the control group (*P* < 0.001 and *P* < 0.05, respectively; Figure 3A, Supplementary Figure 1). The active MMP-2 gelatinolytic activity was augmented in midluteal phase tissues treated for 24 h with CAT compared to the control group (*P* < 0.05; Figure 3B). At 48 h, in midluteal phase, CAT treatment increased active MMP-2 gelatinolytic activity comparing to INH-treated group (*P* < 0.05; Figure 3B, Supplementary Figure 1).

The gelatinolytic activity of MMP-9 active form was detected in both estrous cycle phases, but only at 48-h treatment (Figures 3C,D). In follicular phase explants, treated with CAT, the active MMP-9 gelatinolytic activity increased comparing to the control group (*P* < 0.05; Figure 3C) and was reduced in CAT+INH–treated tissues, in comparison to the respective group treated with CAT (*P* < 0.05; Figure 3C, Supplementary Figure 1).

The gelatinolytic activity of pro–MMP-2 enzyme in the INH-treated group was downregulated at 24 h in follicular phase explants (Figure 3A, Supplementary Figure 1). The stimulatory effect of CAT was higher in follicular phase at 24 h than at 48 h on active MMP-2 gelatinolytic activity (Figure 3A), and the combination of CAT and INH reduced the gelatinolytic activity at 48 h comparing to 24-h treatment (Figure 3A, Supplementary Figure 1). In midluteal phase endometrium, all treatments upregulated the active gelatinolytic activity of MMP-2 at 24 h, compared to 48 h (Figure 3B, Supplementary Figure 1).

## Discussion

In the present study, CAT induced COL1 expression in explants of mare endometrium, at follicular phase and midluteal phase in a time-dependent manner. The *COL1A2* mRNA results show that CAT acts as a profibrotic protease, mainly in follicular phase, as a response to a shorter stimulus, and in midluteal phase as a response to a longer stimulus. During the follicular phase, endogenous estrogen thickens the uterine wall and increases uterine muscular tone and vascularization. The cervix is relaxed and opens (50). The endometrial glands also proliferate, and the lamina propria becomes highly edematous (10). The mare endometrium is more prone to inflammation and more reactive at estrus, which might explain why the explants obtained at the follicular phase, under the influence of estrogens, were reactive to CAT after a short time of stimulation. Moreover, a longer time of CAT exposition was needed to increase expression of COL1 at the protein level. The COL1 protein relative abundance was increased by CAT only at 48 h in both estrous cycle phases.

One of the aims of this study was to evaluate if by inhibiting CAT using a specific inhibitor (IHN) it would be possible to reduce CAT-induced COL1 relative abundance in equine endometrium. This inhibitor blocks the increase of monocyte chemoattractant protein 1 and tumor necrosis factor α, both linked to airway hyperactivity (29), and blocks neutrophilia (51). We showed in our study that the inhibitory effects of IHN were detected in the longest treatment time, corresponding to the increased COL1 relative abundance induced by CAT treatment. To the best of our knowledge, this is the first study describing that by inhibiting CAT, it is possible to reduce COL1 relative abundance in equine endometrium *in vitro*. Therefore, we suggest that this treatment could be a possible approach to prevent the formation of endometrosis. In fact, INH offers a promising therapeutic strategy in chronic inflammatory conditions, such as asthma or COPD (26). Future *in vivo* studies are crucial to test this hypothesis. Currently, despite the therapies proposed to treat equine endometrosis, there is no routinely available effective treatment (42, 52). Several therapeutic approaches, as mechanical curettage or intrauterine application of chemical agents (kerosene, dimethyl sulfoxide, isotonic salt) or mesenchymal stem cells, have been studied (53–55). Nevertheless, they caused rather short-term beneficial effects and/or did not improve pregnancy rates (53–55). Thus, the need for evaluating the *in vivo* efficacy of INH in the treatment of equine endometrosis associated with NETs is imperative. Indeed, our findings may be the grounds for further *in vivo* trials for INH testing.

Fibrosis is the result of a disruption in the balance of the extracellular matrix, with increased synthesis and deposition of extracellular matrix components and decreased degradation of those products (56). The MMPs have been considered as being part of the highly regulated systems that control this extracellular matrix turnover (57). An increase in the active form of MMP-2 has been reported in mare endometrosis (58), although other works showed no changes in MMP-2 or MMP-9 expression between normal and fibrotic equine endometrium (59). Another study done by Centeno et al. (60) found that *MMP2* transcription was upregulated in endometrial fibrosis. Moreover, we have recently reported an upregulation of MMP-2 and MMP-9 levels in mare endometrial tissue with mild to moderate lesions, as well as an increase of MMP-9 levels in fibroblasts and epithelial cells challenged by TGFβ1 (35). In other tissues, CAT has previously been capable to activate pro–MMP-2 in human tumor cell invasion (61) and together with MMP-9 may enhance TGFβ signaling in a tumor murine model (62). The inconsistency between MMP expression found in normal and fibrotic equine endometrium may be explained by the fact that fibrotic changes, as in other tissues (e.g., lungs) (63), are diffuse. The collected tissue may not always reflect the entire condition of the fibrotic organ and thus might not fully address the cellular and spatial heterogeneity of fibrosis. Additionally, because endometria at different stages of fibrosis were obtained postmortem from different mares, it was not feasible to evaluate the evolution of the fibrogenic process individually. This may have affected the results and thus could also explain the inconsistent pattern found. However, despite these limitations, also observed in other tissues, understanding of the molecular pathways and the expression of various factors involved in equine endometrosis is rather important, by unraveling changes associated with this pathological condition.

In our study, the gelatinolytic activity of MMP-2 active form in endometrial explants increased in response to CAT treatment after the shortest treatment time (24 h), at both estrous cycle phases. Nevertheless, this profibrotic effect of CAT was diminished with INH addition in follicular phase tissue treated for 24 h. Apparently, MMP-2 appears to be involved in an immediate response, perturbing extracellular matrix balance. So, MMP-2 can mediate an acute response to a CAT-induced inflammation, regardless of the estrous cycle phase.

In follicular phase endometrial explants, the gelatinolytic activity of MMP-9 active form increased with CAT treatment and was inhibited by INH at 48 h. This suggests MMP-9 involvement, especially in follicular phase equine endometrium, remodeling the fibrogenic response to a prolonged exposition to CAT.

Elevated levels of MMPs in the endometrium may also indicate a cellular response to an altered extracellular matrix balance, as part of the normal regulation of MMP expression. In fact, the main role attributed to MMPs is their action on the turnover and degradation of extracellular matrix substrates. Regulation of MMPs activity takes place at the stages of gene transcription, protein production, activation of proenzymes, and inhibition of the active enzymes by tissue inhibitor of matrix metallopeptidases or α2-macroglobulin (64). Many of these factors can contribute to the differences found between gene transcription, proenzyme, and active form of MMP-2 and MMP-9. In addition, MMP-9 may be regulated by ovarian steroids, which can explain why this enzyme activity differed according to various estrous cycle phases (65). Many mechanisms are involved in the response to CAT profibrotic stimulus, and more studies are necessary to unravel the role of MMPs, either in healthy or fibrotic endometrium.

## Conclusions

Even though our previous (18, 20) and present results suggest that ELA and CAT are profibrotic factors and are involved in equine endometrial fibrosis establishment, the study of other causes, including the role of other proteases found in NETs, is vital to fully understand the mechanisms of endometrosis pathogenesis. The use of a selective CAT inhibitor was effective on the reduction of COL1 expression. Therefore, these novel data may contribute to the development of a new prophylactic or therapeutic approach for endometrosis. Although the use of a broad-spectrum protease inhibitor or specific selective inhibitors combined may be needed to obtain a strong and more effective inhibitory effect. MMP-2 might be involved in an earlier response to CAT, independent of estrous cycle phase, and MMP-9 in a later response, mainly in the follicular phase.

## Data Availability Statement

The raw data supporting the conclusions of this article will be made available by the authors, without undue reservation.

## Author Contributions

GF-D and DS: conceptualization, resources, data curation, visualization, supervision, project administration, and funding acquisition. AA, CF, SM, MR, AS-M, KL, and BG-K: methodology. AA, CF, and LT: formal analysis. AA, CF, MR, SM, AS-M, and KL: investigation. AA: writing—original draft preparation. GF-D, DS, AS-M, MR, BG-K, and LT: writing—review and editing. All authors have read and agreed to the published version of the manuscript.

## Conflict of Interest

The authors declare that the research was conducted in the absence of any commercial or financial relationships that could be construed as a potential conflict of interest.
