# Supplementary material for: The Inhibition of Cathepsin G on Endometrial Explants With Endometrosis in the Mare
Source: Front Vet Sci. 2020 Oct 30;7:582211. doi: 10.3389/fvets.2020.582211 (PMC7661753; doi:10.3389/fvets.2020.582211)
Supplement: Supplementary file 1 [file Data_Sheet_1.docx]

Supplementary Material

**Supplementary Table S1**: The effect of oxytocin (OXT, 10^−7^ M) on prostaglandin (PG) F_2α_ secretion from equine endometrial explants in follicular phase (FP) and mid-luteal phase (MLP) treated for 24 h or 48 h, relative to control (non-treated explants). Results are presented as means ± SEM. Different superscript letters indicate statistical differences between respective columns (within estrous cycle phases and times of treatment). The results were considered significant at P < 0.05.

| **Estrous Cycle Phase** | **FP** | | | | **MLP** | | | |
| --- | --- | --- | --- | --- | --- | --- | --- | --- |
| **Time of Treatment** | **24 h** | | **48 h** | | **24 h** | | **48 h** | |
| **Treatment** | **Control** | **OXT**  **(**10^−7^ M**)** | **Control** | **OXT**  **(**10^−7^ M**)** | **Control** | **OXT**  **(**10^−7^M**)** | **Control** | **OXT (**10^−7^ M**)** |
| PGF_2α_ secretion (ng/mg) | 4.43 ± 1.3 ^a^ | 7.71 ±  2.8 ^b^ | 6.30 ± 1.3 ^a^ | 13.9 ±  6.2 ^b^ | 8.50 ± 1.6 ^a^ | 13.2 ±  2.3 ^b^ | 6.4 ±  0.9 ^a^ | 11.0 ± 1.5 ^b^ |

**Supplementary Table S2**: Levels of significance (P values) for 2-, 3- and 4-way interactions between estrous cycle phases, treatment time, and cathepsin G (CAT) or Cathepsin G Inhibitor I (INH) treatments in the analyses of relative transcript of target genes, COL1 protein relative abundance and gelatinolytic activity of MMP-2 and -9. The results were considered significant at *P* < 0.05 and are highlighted in yellow color.

| Interaction | *COL1A2* | COL1 | *MMP2* | Pro-MMP-2 | Active MMP-2 | *MMP9* | Pro-MMP-9 | Active MMP-9 |
| --- | --- | --- | --- | --- | --- | --- | --- | --- |
| CAT x INH | 0.0002 | 0.0237 | 0.0002 | 0.0989 | 0.2229 | 0.0246 | 0.522 | 0.0484 |
| CAT x treatment time | 0.8625 | 0.0247 | 0.0034 | 0.5608 | 0.0029 | 0.583 | 0.9213 | . |
| CAT x estrous cycle phase | 0.0282 | 0.9823 | 0.887 | 0.2488 | 0.4438 | 0.417 | 0.6549 | 0.7101 |
| INH x treatment time | 0.5519 | <.0001 | 0.4835 | 0.2351 | 0.5119 | 0.0525 | 0.7105 | . |
| INH x estrous cycle phase | 0.5877 | 0.2437 | 0.0438 | 0.3968 | 0.0987 | 0.409 | 0.8159 | 0.5089 |
| Time of treatment x estrous cycle phase | 0.0002 | 0.0711 | 0.2474 | 0.2057 | 0.7389 | 0.0079 | 0.2558 | . |
| CAT x INH x treatment time | 0.5701 | 0.0161 | 0.4812 | 0.4717 | 0.0141 | 0.6212 | 0.3652 | . |
| CAT x INH x estrous cycle phase | 0.1657 | 0.1039 | 0.1516 | 0.3425 | 0.2843 | 0.8317 | 0.755 | 0.1491 |
| CAT x treatment time x estrous cycle phase | 0.0085 | 0.6088 | 0.4743 | 0.8015 | 0.0926 | 0.291 | 0.7341 | . |
| INH x treatment time x estrous cycle phase | 0.9312 | 0.2582 | 0.694 | 0.7429 | 0.0478 | 0.611 | 0.2875 | . |
| CAT x INH x treatment time x estrous cycle phase | 0.0365 | 0.944 | 0.3722 | 0.4534 | 0.645 | 0.4925 | 0.7734 | . |

Abbreviations: *COL1A2* - collagen type 1 α2; COL1 – collagen type I protein; *MMP2* - matrix metallopeptidase 2; *MMP9* - matrix metallopeptidase 9

**Supplementary Table S3**: Listed significant differences of the same treatments between the follicular phase (FP) and mid-luteal phase (MLP) of the estrous cycle, within each treatment time.

| **Evaluated variables** | **Treatment comparison** | **P value** | **Figures** |
| --- | --- | --- | --- |
| *COL1A2* transcription | CAT 48h FP *vs* CAT 48h MLP | *P* < 0.0001 | 1A, 1B |
|  | CAT + INH 48h FP *vs* CAT + INH 48h MLP | *P* < 0.05 |  |
| COL1 protein relative abundance | CAT + INH 48h FP *vs* CAT + INH 48h MLP | *P* < 0.05 | 1C, 1D |
| *MMP9* transcription | CAT + INH 48h FP *vs* CAT + INH 48h MLP | *P* < 0.05 | 2C, 2D |
| Pro-MMP-2 activity | CAT 24h FP *vs* CAT 24h MLP | *P* < 0.05 | 3A, 3B |
| Active MMP-2 activity | CAT 48h FP *vs* CAT 48h MLP | *P* < 0.01 | 3A, 3B |

*COL1A2* - collagen type 1 α2; COL 1 – collagen type I; *MMP2* - matrix metallopeptidase 2; *MMP9* - matrix metallopeptidase 9; CAT – cathepsin G; INH – Cathepsin Inhibitor I; FP – follicular phase; MLP – mid-luteal phase.

**Supplementary Fig. S1**

| **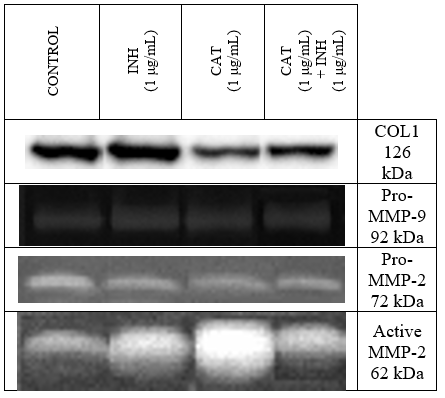** | 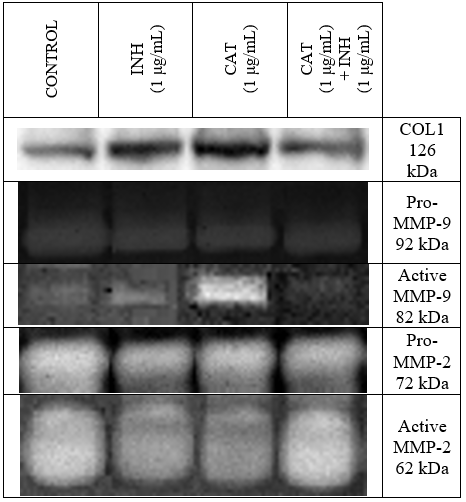 |
| --- | --- |
| **A** – 24h FP endometrium explant | **B** – 48h FP endometrium explant |
| **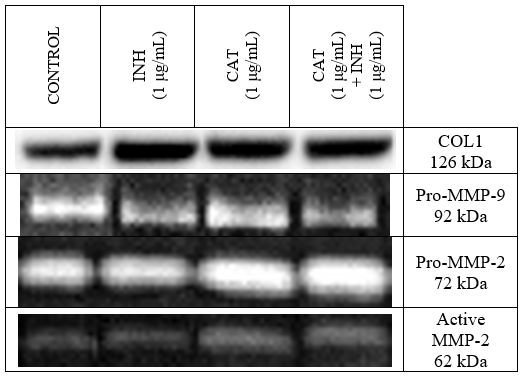** | 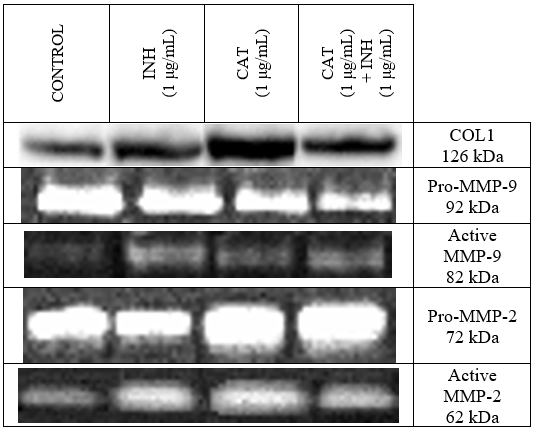 |
| **C** – 24h MLP endometrium explant | **D** – 48h MLP endometrium explant |

**Supplementary Fig. S1:** Representative panels of type I collagen (COL1) western blotting and pro and active form of MMP-2 and MMP-9 zymograms in mare endometrium in follicular phase (FP) or mid-luteal phase (MLP) treated for 24h or 48h with cathepsin G (CAT 1 μg/mL), CAT inhibitor I (INH; 1µg/mL) and CAT (1µg/mL) + INH (1µg/mL). **A** - 24h treatment of FP endometrium explants; **B** – 48h treatment of FP endometrium explants, **C** – 24h treatment of MLP endometrium explants; and **D** – 48h treatment of MLP endometrium explants.
